# Climate factors and gestational diabetes mellitus risk – a systematic review

**DOI:** 10.1186/s12940-020-00668-w

**Published:** 2020-11-09

**Authors:** Emma V. Preston, Claudia Eberle, Florence M. Brown, Tamarra James-Todd

**Affiliations:** 1 Department of Environmental Health, Harvard T.H. Chan School of Public Health, Building 1, Room 1411, 677 Huntington Ave, Boston, MA 02118 USA; 2 Medicine with specialization in Internal Medicine and General Medicine, Hochschule Fulda - University of Applied Sciences, Fulda, Germany; 3 Adult Diabetes Section, Joslin Diabetes Center, Boston, MA USA; 4 Department of Epidemiology, Harvard T.H. Chan School of Public Health, Boston, MA USA

**Keywords:** Climate, Season, Temperature, Gestational diabetes, Pregnancy

## Abstract

**Background:**

Current and projected increases in global temperatures and extreme climate events have led to heightened interest in the impact of climate factors (i.e. ambient temperature, season/seasonality, and humidity) on human health. There is growing evidence that climate factors may impact metabolic function, including insulin sensitivity. Gestational diabetes mellitus (GDM) is a common pregnancy complication, with an estimated global prevalence of up to 14%. While lifestyle and genetic risk factors for GDM are well established, environmental factors may also contribute to GDM risk. Previous reviews have summarized the growing evidence of environmental risk factors for GDM including endocrine disrupting chemicals and ambient air pollution. However, studies of the effects of climate factors on GDM risk have not been systematically evaluated. Therefore, we conducted a systematic review to summarize and evaluate the current literature on the associations of climate factors with GDM risk.

**Methods:**

We conducted systematic searches in PubMed and EMBASE databases for original research articles on associations of climate factors (i.e. ambient temperature, season/seasonality, and humidity) with GDM and/or related glycemic outcomes for all publication dates through September 20th, 2020.

**Results:**

Our search identified 16 articles on the associations of ambient temperature and/or season with GDM and maternal glycemic outcomes during pregnancy, which were included in this review. Despite inconsistencies in exposure and outcome assessment, we found consistent evidence of a seasonal effect on GDM risk, with higher prevalence of GDM and higher pregnancy glucose levels in summer months. We found suggestive evidence of an association between higher ambient temperature and elevated glucose levels from GDM screening tests.

**Conclusion:**

Climate factors may be associated with GDM risk. However, further research is needed to evaluate these associations and to elucidate the specific mechanisms involved.

## Background

Increased awareness of global climate change has led to heightened interest in the impact of climatic factors on human health [1]. Historically, much of the research on the human health effects of climate change focused on extreme temperature events and infectious diseases [2–4]. However, there is growing evidence indicating that climate factors (i.e. season, ambient temperature, and humidity) may alter metabolic function, including insulin sensitivity [1, 5]. The insulin resistant state of pregnancy may be particularly sensitive to climate factors, which could impact the body’s metabolism, increasing gestational diabetes (GDM) risk. Thus, it is important to review the state of the literature on climate factors and GDM.

GDM, defined as glucose intolerance that is less than overt diabetes first occurring during pregnancy, is one of the most common pregnancy complications [6]. The prevalence of GDM has increased over the past 20 years and is currently estimated to affect up to 14% of pregnancies worldwide [7], though estimates vary based on the population and diagnostic criteria [6, 8–10]. GDM is associated with both short- and long-term adverse pregnancy, maternal and neonatal outcomes, including preeclampsia, cesarean section, preterm birth, macrosomia and neonatal hypoglycemia [7, 11]. Mothers are at increased risk of developing type 2 diabetes [12–14] and cardiovascular disease [15, 16] in later life. Recent studies demonstrate that offspring may be at increased risk of higher adiposity and abnormal glucose metabolism in mid-childhood [6, 11, 17].

Lifestyle and genetic factors such as diet, body mass index (BMI), physical activity, parity, age, and family history may not fully account for GDM risk [18–20]. Recent reviews have examined the potential role of environmental exposures including endocrine disrupting chemicals [21, 22], toxic metals [21], and air pollution [23, 24] in the development of GDM. However, climate factors may also play a role in GDM risk [25–27]. With the current and projected rise in global surface temperatures and increased frequency of extreme weather events [28], we aim to review the role of climate factors on this significant pregnancy complication.

### Rationale for climate factors as a risk factor for gestational diabetes

Previous meta-analyses and primary research studies have found associations between ambient temperature, season, and humidity with type 1 and/or type 2 diabetes [29–31]. Potential physiologic explanations for these associations include several different mechanisms, which have also been discussed as possible mechanisms for associations between climate factors and GDM. Cold ambient temperature may cause activation of brown adipose tissue leading to improved insulin sensitivity [32]. On the other hand, high ambient temperatures and relative humidity could cause dehydration and result in hemoconcentration during the summer, leading to spurious increased blood glucose concentrations [33]. Seasonal fluctuations in serum vitamin D levels could also contribute to the observed association between season and type 2 diabetes risk [34]. In fact, vitamin D may aid in glucose regulation by increasing insulin secretion and sensitivity, aiding in beta-cell function, and decreasing systemic inflammation [35]. Alternatively, changes in diet and physical activity levels related to ambient temperature, season, and humidity may contribute to observed seasonal fluctuations in diabetes risk.

A growing number of studies have evaluated climate factors as they relate to GDM; however, these studies have not been systematically reviewed. Therefore, we conducted a systematic review of epidemiological studies evaluating the associations of climate factors with GDM and glycemic outcomes during pregnancy, to assess how climate factors were associated with the continuum of glucose intolerance. We evaluated epidemiological studies identified in PubMed and EMBASE, summarized the state of the literature, identified research gaps, and suggested key next steps for future studies of the influence of climate factors on GDM. To our knowledge, this is the first systematic review of climate factors as they relate to GDM.

## Methods

### Search strategy

We conducted a systematic literature search in PubMed and EMBASE databases for original research articles for all publication dates through September 20th, 2020, based on the guidelines of the PRISMA statement [36]. We considered original epidemiologic studies in pregnant women written in the English language with available full-text. Studies were included in the review if they related one or more climate factors to one or more glucose-related outcomes during pregnancy. Maternal glycemic outcomes included gestational glucose levels, markers of insulin sensitivity, beta cell function, categories of glucose tolerance derived from glucose challenge test (GCT) and/or oral glucose challenge test (OGTT) screenings (e.g. abnormal GCT or OGTT values), or GDM diagnosis.

In PubMed we searched using the following combination of Medical Subject Headings (MeSH) terms and freetext title or abstract terms (tiab): ((diabetes, gestational [MeSH] OR gestational diabetes [tiab]) OR ((Diabetes Mellitus [Mesh:NoExp] OR glucose tolerance test [MeSH] OR blood glucose [MeSH] OR hyperglycemia [MeSH] OR diabetes [tiab] OR diabetic [tiab] OR blood glucose [tiab] OR glucose intolerance [tiab] OR glucose tolerance [tiab] OR hyperglycemia [tiab]) AND (Pregnancy [Mesh:NoExp] OR Pregnancy Outcome [Mesh:NoExp] OR Pregnancy, High-Risk [Mesh] OR Pregnancy Complications [Mesh:NoExp] OR pregnanc*[tiab] OR pregnant [tiab]))) AND (climate [MeSH] OR weather [MeSH] OR climate [tiab] OR seasonality [tiab] OR season [tiab] OR ambient temperature [tiab] or dew point [tiab] OR humidity [tiab]). We restricted our PubMEd search using the following search terms: NOT (Animals [MeSH] NOT Humans [MeSH]) NOT (Case Reports [ptyp] OR Comment [sb] OR Editorial [ptyp] OR Guideline [ptyp] OR Letter [ptyp] OR News [ptyp] OR Practice Guideline [ptyp]). In EMBASE, we searched using the following combination of Emtree and text search terms: (‘pregnancy diabetes mellitus’/exp. OR ‘gestational diabetes’:ti,ab,kw) OR ((diabetes:ti,ab,kw OR ‘glucose tolerance’:ti,ab,kw OR ‘glucose intolerance’:ti,ab,kw OR hyperglycemia:ti,ab,kw OR ‘blood glucose’:ti,ab,kw OR ‘diabetes mellitus’/de OR ‘impaired glucose tolerance’/de OR ‘hyperglycemia’/de) AND (‘pregnant woman’/exp. OR ‘pregnancy’/exp. OR pregnancy:ti,ab,kw OR ‘pregnant women’:ti,ab,kw)) AND (‘seasonal variation’:ti,ab,kw OR season:ti,ab,kw OR ‘ambient temperature’:ti,ab,kw OR ‘environmental temperature’:ti,ab,kw OR weather:ti,ab,kw OR climate:ti,ab,kw OR ‘climate change’:ti,ab,kw OR ‘climate’/exp. OR ‘meteorological phenomena’/exp. OR ‘environmental temperature’/exp. OR ‘seasonal variation’/exp). In EMBASE, we used the following search restrictions: AND ([article]/lim OR [article in press]/lim) AND [humans]/lim AND [english]/lim.

**Fig. 1:**
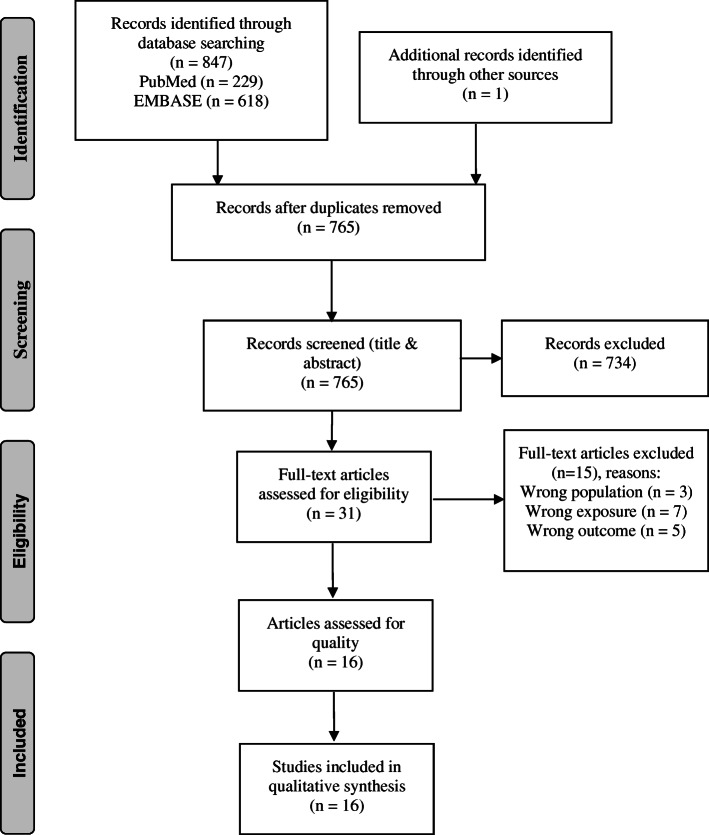
PRISMA flow diagram illustrating the selection process for studies included in this review [adapted from Moher et al. 2009 [36]]

Articles were excluded if they were not available in English, were duplicates, conducted in animals, had irrelevant exposures, or irrelevant outcomes, were irrelevant study types (i.e. case-reports, reviews, meta-analyses), were not in pregnant women, or the study outcomes occurred outside of pregnancy (e.g. postpartum glucose levels) (see Fig. 1). In addition to our database searches, we manually checked the listed references of identified articles and relevant reviews for additional articles that we may have missed in our search in order to maximize the likelihood of identifying all relevant articles.

## Results

Our systematic search strategy returned 847 articles, 229 from PubMed and 618 from EMBASE (Fig. 1). We identified one additional article by reviewing article reference lists. After excluding duplicate articles (*n* = 83), we excluded an additional 734 articles through title and abstract screening. We included 31 articles in our full text review, and excluded an additional 15 based on our inclusion/exclusion criteria (non-pregnant population: *n* = 3, wrong exposure: *n* = 7, wrong outcome: *n* = 5). We did not find any articles on humidity and GDM or maternal glycemic outcomes. Additionally, our search did not identify any articles that investigated other maternal or neonatal short and long-term health outcomes as consequences of GDM and/or glycemic outcomes during pregnancy associated with climate factors. In total, we included 16 articles on the associations of ambient temperature and/or season with GDM (Table 2) and maternal glycemic outcomes (Table 3) during pregnancy in this review.

### Glucose related study outcomes

There was significant heterogeneity in method of GDM screening/diagnosis across the included studies. Table 1 summarizes the GDM screening methods and diagnostic criteria used by the included studies. The majority of screening methods fall into two categories: (1) a one-step approach, where GDM is diagnosed based on results of a single fasting oral glucose tolerance test (OGTT), or (2) a two-step approach, where women are pre-screened, generally with a non-fasting glucose challenge test (GCT), and only women with abnormal GCT glucose levels (e.g. > 140 mg/dL) receive a subsequent OGTT to diagnose GDM. The International Association of Diabetes in Pregnancy Study Group (IADPSG) criteria follows a one-step approach using a fasting 2-h 75-g OGTT with relatively low glucose level thresholds, requiring only one abnormal value for a positive test. Many countries and organizations, including the WHO, have adopted the IADPSG criteria. Conversely, in the United States, GDM is generally screened and diagnosed using a more conservative two-step approach, consisting of [1] a non-fasting 50-g GCT, followed by [2] a fasting 3-h 100-g OGTT, with higher glucose level thresholds than the IADPSG criteria, requiring two abnormal values for a positive test [8]. Differences in GDM screening and diagnostic criteria are important factors to consider when comparing study results below.

**Table 1:** Gestational diabetes mellitus screening and diagnostic criteria

Guidelines	Year	Approach	GCT	GCT Glucose Threshold	OGTT	OGTT Glucose Threshold Values				GDM Diagnosis
						Fasting	1-h	2-h	3-h	
**One-step approaches**										
International Association of Diabetes and Pregnancy Study Group (IADPSG) [48]	2010	1-step	–	–	75-g	5.1 mmol/L (92 mg/dL)	10 mmol/L (180 mg/dL)	8.5 mmol/L (153 mg/dL)	–	≥1 abnormal OGTT
World Health Organization (WHO) [49]	1999	1-step	–	–	75-g	7 mmol/L (126 mg/dL)	–	7.8 mmol/L (140 mg/dL)	–	≥1 abnormal OGTT
WHO - Modified [45, 50]	2006	1-step	–	–	75-g	DIP: 7 mmol/L (126 mg/dL); GDM: 5.1–6.9 mmol/L (92–124 mg/dL)		DIP: 11.1 mmol/L (200 mg/dL); GDM: 8.5–11 mmol/L (153–198 mg/dL)		DIP or GDM: ≥1 abnormal OGTT
**Two-step approaches**										
Australian Diabetes in Pregnancy Society (ADIPS) [51]	1991	2-step	50-g	7.8 mmol/L (140 mg/dL)	75-g	5.5 mmol/L (99 mg/dL)	–	8 mmol/L (144 mg/dL)	–	≥1 abnormal OGTT
Carpenter and Coustan [52]	1982	2-step	50-g	7.8 mmol/L (140 mg/dL)	100-g	5.3 mmol/L (95 mg/dL)	10 mmol/L (180 mg/dL)	8.6 mmol/L (155 mg/dL)	7.8 mmol/L (140 mg/dL)	≥2 abnormal OGTT
National Diabetes Data Group (NDDG) Criteria [53]	1979	2-step	50-g	7.8 mmol/L (140 mg/dL)	100-g	5.8 mmol/L (105 mg/dL)	10.5 mmol/L (189 mg/dL)	9.2 mmol/L (166 mg/dL)	8.0 mmol/L (144 mg/dL)	≥2 abnormal OGTT
WHO - Modified [46, 49]	1999	2-step	Random glucose	6.5 mmol/L (117 mg/dL)	75-g	6 mmol/ (108 mg/dL)	–	7.8 mmol/L (140 mg/dL)	–	≥1 abnormal OGTT
WHO - Modified [37]	1999	2-step	50-g	7.7 mmol/L (139 mg/dL)	75-g	6.1 mmol/L	–	7.8 mmol/L	–	≥1 abnormal OGTT

*Abbreviations*: *GCT* glucose challenge test, *OGT* oral glucose tolerance test, *GDM* gestational diabetes mellitus, *DIP* diabetes in pregnancy

In addition to GDM diagnosis, we included studies with maternal glycemic outcomes such as additional categories of glucose intolerance based on GCT and/or OGTT screening results [e.g. abnormal GCT (> 140 mg/dL), or hyperglycemia in pregnancy]. As with GDM, the definitions and thresholds used in these classifications varied across studies. Multiple studies assessed glucose levels, analyzed as continuous outcomes, usually from GCT and/or OGTT screenings. Two studies also assessed various markers of insulin resistance (IR) [e.g. Matsuda index and homeostasis model assessment of insulin (HOMA-IR, HOMA S)] and beta cell function [e.g. insulin sensitivity index 2 (ISSI-2), insulinogenic index (IGI)/HOMA-IR, and HOMA B].

Timing of outcome assessment also varied by study (Tables 2 & 3), with GDM screening and glycemic outcome measurement typically occurring between 24 and 28 weeks gestation, but as early as 16–18 weeks in some studies [42]. Differences in outcome assessment could also impact observed associations with climate factors as well as variability across studies.

**Table 2:** Climate factors and GDM study table

Author, Year	Study size & location	Study characteristics	Age range or Mean ± SD	Exposure measures	GDM screening & diagnostic criteria	Model covariates	Main findings
Meek, 2020 [37]	*n* = 23,375 United Kingdom	Cohort: enrolled pregnant women with singleton pregnancies from 1/2004 to 12/2008	30.7 ± 5.6 years	A) Season: day of delivery B) Ambient temperature (°C): daily mean temp on day of screening	2-step approach: 1) 50-g GCT at 28 weeks, if > 7.7 mmol/l 2) 75-g OGTT OGTT criteria: WHO 1999 (1/2004–8/2007) Modified WHO 1999 (8/2007–12/2008)	Maternal age, BMI, parity, ethnicity	•GDM incidence varied significantly by day of glucose screening throughout the year (*p* = 0.031). GDM incidence was highest in births during Sept/Oct and lowest in births during March •Daily mean temperature on day of GCT screening were associated with increased risk of abnormal GCT (OR 1.21, 95% CI: 1.10, 1.32 per 5 °C increase) and increased odds of GDM (OR 1.13, 95% CI: 1.02, 1.25 per 5 °C increase)
Molina-Vega, 2020 [38]	*n* = 2366 Malaga, Spain	Cohort: retrospective cohort of women referred to a Pregnancy and Diabetes clinic for GDM screening	32 ± 5.2 years	A) Ambient temperature: 1) mean ∆ temp 2) mean temp Day of OGTT, 14 days pre-OGTT, and 28 days pre-OGTT B) Season Winter (Dec 21st – Mar 20th) Spring (Mar 21st – Jun 20th) Summer (Jun 21st – Sep 20th) Autumn (Sep 21st – Dec 20th)	NDDG criteria	Maternal age	•Odds of GDM were highest in summer (OR 1.78 CI: 1.34, 2.37) compared to autumn •Higher mean temperature on the day of OGTT screening and 14- and 28-days pre-OGTT were associated with increased risk of GDM diagnosis (e.g. Mean temp on day of OGTT: OR 1.03, 95% CI: 1.01, 1.05) •When stratified, these associations were only present in the seasons where temperatures were increasing (Mar-Aug)
Su, 2020 [21]	*n* = 371,131 Taiwan	Cohort: population-based cohort study of pregnant women with deliveries between 2013 and 2014 in Taiwan	Not provided	A) Season B) Ambient temperature (°C): 1) mean temp 2) daily temp ∆. Mean temperature: day of OGTT, 7, 14, 21, 28, 35 days pre-OGTT. Temperature ∆: daily difference between min and max temp on OGTT day and average ∆ 7, 14, 21, 28, 35 days pre-OGTT	IADPSG & Carpenter and Coustan criteria	Maternal age	•Age-adjusted odds of GDM were highest is summer (OR 1.05, 95% CI: 1.04, 1.07) and fall (OR 1.04, 95% CI: 1.02, 1.06) compared to winter •Increased mean daily temperature (per 1 °C increase) was associated with increased age-adjusted odds of GDM for mean temperatures between 14 and 17 °C (OR 1.03, 95% CI: 1.02, 1.03) and even more strongly for temperatures between 28 and 30 °C (OR 1.54, 95% CI: 1.48, 1.60) •Increased daily temperature difference (per 1 °C increase) was associated with lower odds of GDM (OR 0.90, 95% CI: 0.87, 0.92)
Petry, 2019 [39]	*n* = 1074. Cambridge, United Kingdom	Cohort: Cambridge Baby Growth Study, enrolled pregnant women during early pregnancy between 4/2001–3/2009	33.4 years	Season: Winter (Dec-Feb) Spring (Mar-May) Summer (Jun-Aug) Autumn (Sep-Nov)	IADPSG (based on fasting and 1 h only)		•Season of OGTT was not associated with GDM
Shen, 2019 [40]	*n* = 2120. Brisbane & Newcastle, Australia	Cohort: Women enrolled at Australian sites of the Hyperglycemia and Adverse Pregnancy Outcome (HAPO) from 2001 to 2006	29.6 ± 5.4 years	Season: Winter (Jun-Aug) Spring (Sep-Nov) Summer (Dec-Feb) Autumn (Mar-May)	WHO criteria		•No significant difference in GDM prevalence by season
Retnakaran, 2018 [41]	*n* = 1464. Toronto, Canada	Cohort: enrolled pregnant women at time of GDM screening	34 ± 4 years	Ambient temperature (°C): 1) mean temp 2) daily temp change. Mean temperature: day of OGTT, 7, 14, 21, 28, 35, 42, 49, 56 days pre-OGTT. Temperature ∆: daily difference between min and max temp on OGTT day and average ∆ 7, 14, 21, 28, 35, 42, 49, 56 days pre-OGTT	NDDG criteria (All women received OGTT)	Maternal age, ethnicity, FH of diabetes, pp-BMI, GWG up to OGTT, weeks gestation at OGTT	•Temperature ∆ was associated with increased risk of GDM, only in the season where daily temperature was increasing •For example, in Feb-July temperature ∆ in the preceding 14 days was associated with GDM (OR 1.20, 95% CI: 1.05, 1.37)
Vasileiou, 2018 [27]	A) *n* = 7618 B) *n* = 768 Athens, Greece	Two cohorts: A) Retrospective cohort: pregnant women who underwent a 100 g OGTT from 2002 to 2012. B) Prospective cohort: pregnant women enrolled in 3rd trimester followed over 18 month period from 1/2013–6/2014.	Not provided	A) Season B) Ambient Temperature: 1) Mean monthly temperature 2) Daily temperature @ 9 am Three temp groups: 1) < 24.9 °C 2) 25–29.9 °C 3) > 30 °C	Study A: Carpenter and Coustan criteria Study B: IADPSG criteria	Unadjusted	Study A: •Odds of GDM were significantly higher in summer compared to winter (OR 1.65. 95% CI: 1.43, 1.90) Study B: •Temperature was not associated with GDM
Chiefari, 2017 [42]	*n* = 5473, Calabria, Italy	Cohort: Study population formed based on women who underwent an OGTT for GDM screening at a hospital in Calabria, Italy from 8/2011–12/2016.	33 (29–36) years	Seasons: Fall, Winter, Spring, Summer, Warm half & cold half of the year	IADPSG criteria	Unadjusted	•GDM incidence was significantly higher in summer (33.7%) and lower in the winter (23.3%) compared to the spring and fall •GDM incidence was significantly lower in the cold (< 15 °C; 24.2%) compared to warm (> 15 °C; 31.4%) half of the year
Booth, 2017 [43]	*n* = 555,911, Toronto, Canada	Cohort: study population formed of births in greater Toronto area from 4/1/2002–3/31/2014 from administrative health databases.	30.9 ± 5.4 years	Ambient temperature: Average temperature 30-days pre-GDM screening (27 weeks)	ICD-10-CA codes (E10, E11, E13, E14, O24) or ≥ 2 diabetes insurance claims in the last 120 days of pregnancy	Maternal age, parity, neighborhood income, world region, year	•Significant association between higher ambient temperature and greater odds of GDM •Each 10 °C increase in mean 30-day temp associated with a 6% increased odds of GDM (OR 1.06, 95% CI: 1.04–1.07)
Katsarou, 2016 [25]	*n* = 11,538, Skane county, Sweden	Cohort: Mamma Study, recruited women from 4 obstetric delivery departments in Skane county, Sweden from 2003 to 2005.	29.9 ± 5.1 years	Seasons: Winter (Dec-Feb) Spring (Mar-May) Summer (June-Aug) Fall (Sept-Nov) Mean monthly ambient temperature	WHO (1999) criteria, 2 h OGTT threshold	Maternal age	•GDM frequency differed significantly by month and season (highest in June/Summer and lowest in March/Spring) •OGTT during summer was associated with increased frequency of GDM compared to all other seasons (OR 1.51, 95% CI: 1.24–1.83)
Verburg, 2016 [44]	n = 60,30, South Australia	Cohort: women with singleton births from South Australian Perinatal Statistics Collection (SAPSC) data from 2007 to 2011.	< 20 to > 40	Estimated date of conception (eDoC) Based on birth date and gestational age at birth (dating ultrasound and/or LMP) *Note Australian Summer (Dec-Feb) Winter (June-Aug)	ADIPS (1998) criteria	Maternal age, BMI, parity, ethnicity, socioeconomic status, chronic hypertension	•GDM was significantly associated with season of eDoC (*p* < 0.001) •Adjusted incidence of GDM was highest in pregnancies with eDoC in August (6.6%) and lowest in pregnancies with eDoC in January (5.41%)
Moses, 2016 [45]	*n* = 7343, Wollongong, Australia	Cohort: pregnant women with OGTT medical record data during 2012–2014 from both public and private pathology labs in the Wollongong, Australia area.	Not provided	Seasons: Summer (Dec-Feb) Fall, Winter, Spring	Modified WHO (2006) criteria	Unadjusted	•Prevalence of GDM was 28% lower in winter and 29% higher in summer, compared to the overall prevalence (*p* = 0.002)
Janghorbani, 2006 [46]	*n* = 4852, Plymouth, United Kingdom	Cohort: study population based on pregnant women in Plymouth, UK screened for GDM between 1/1996–12/1997 using data from Plymouth Child Health Database and laboratory and midwifery notes.	GDM: 30.9 ± 5.5 years, Non-GDM: 28.1 ± 5.4 years	Month and season	Modified WHO (1999) criteria	Maternal age, random plasma glucose, infant sex	•The prevalence of GDM was highest in June (2.9%) and Spring (2.3%) and lowest in November (1.1%) and Winter(1.4%), but the differences were not statistically significant (*p* = 0.82, month; *p* = 0.41, season)
Moses, 1995 [26]	*n* = 2749, Wollongong, Australia	Cohort: study population based on women with available OGTT data collected from clinics and obstetric offices from 1/1993 to 6/1994.	27 ± 5.1 years	Month & Season: Summer, Fall, Winter, Spring. Mean monthly ambient temperature (measured @ 9 am) *Note Australian Summer (Dec-Feb) Winter (June-Aug)	ADIPS (1991) criteria	Unadjusted	•Month/season and temperature were not associated GDM

*Abbreviations*: *GDM* gestational diabetes mellitus, *OGTT* oral glucose tolerance test, *NDDG* National Diabetes Data Group, *IADPSG* International, *FH* family history, *BMI* body mass index, *pp-BMI* pre-pregnancy BMI, *GWG* gestational weight gain, Association of Diabetes and Pregnancy Study Group, *WHO* World Health Organization, *LMP* last menstrual period, *ADIPS* Australian Diabetes in Pregnancy Society

**Table 3:** Climate factors and glucose related outcomes study table

Author, Year	Study size & location	Study Characteristics	Age range or Mean ± SD	Exposure measures	Outcome measures	Model covariates	Main Findings
Meek, 2020 [37]	*n* = 23,375, United Kingdom	Cohort: enrolled pregnant women with singleton pregnancies from 1/2004 to 12/2008	30.7 ± 5.6 years	Season: day of delivery	Random plasma glucose, Measured at enrollment (11–16 weeks)	Maternal age, BMI, parity, ethnicity	•Random plasma glucose levels varied significantly by season (*p* < 0.001) and were highest in spring (Mar-Apr) and lowest in fall (Aug-Sept)
Molina-Vega, 2020 [38]	*n* = 2366, Malaga, Spain	Cohort: retrospective cohort of women referred to a Pregnancy and Diabetes clinic for GDM screening	32 ± 5.2 years	A) Ambient temperature: 1) mean ∆ temp 2) mean temp Day of OGTT, 14 days pre-OGTT, and 28 days pre-OGTT B) Season Winter (Dec 21st – Mar 20th) Spring (Mar 21st – Jun 20th) Summer (Jun 21st – Sep 20th) Autumn (Sep 21st – Dec 20th)	Glucose levels (OGTT) A) continuousB) abnormal (NDDG cut offs) Two-step: 1) 50 g GLT 2) 100 g OGTT	Maternal age	•Odds of abnormal 1-, 2-, and 3-h OGTT glucose levels were significantly higher in summer compared to autumn (e.g. 2 h OGTT glucose: OR 1.8, 95% CI: 1.4–2.4) •Mean temperature on the day of the OGTT was negatively correlated with fasting glucose (r = − 0.08) but positively correlated with 1-, 2-, and 3-h OGTT glucose levels
Petry, 2019 [39]	*n* = 1074 Cambridge, United Kingdom	Cohort: Cambridge Baby Growth Study, enrolled pregnant women during early pregnancy between 4/2001–3/2009	33.4 years	Season: Winter (Dec-Feb) Spring (Mar-May) Summer (Jun-Aug) Autumn (Sep-Nov)	1) Glucose levels (OGTT) 2) HOMA S & HOMA B 75 g OGTT at median 28.4 weeks		•Season of OGTT was not associated with OGTT glucose levels •Season of OGTT was not associated with HOMA S or HOMA B
Shen, 2019 [40]	*n* = 2120, Brisbane & Newcastle, Australia	Cohort: Women enrolled at Australian sites of the Hyperglycemia and Adverse Pregnancy Outcome (HAPO) from 2001 to 2006	29.6 ± 5.4 years	A) Season: Winter (Jun-Aug) Spring (Sep-Nov) Summer (Dec-Feb) Autumn (Mar-May) B) Ambient temperature (°C): Mean monthly temp	A) Glucose levels (OGTT) B) HbA1C C) HOMA-IR, 75 g OGTT at 24–36 weeks		•Mean fasting glucose, HbA1c, and HOMA-IR levels were lowest in the summer and highest in the winter months •Mean 1 and 2-h OGTT glucose levels were highest in the summer and lowest in the winter •Fasting glucose (r = − 0.145) and HbA1c (r = − 0.069) were negatively correlated with mean monthly temperature •1 h and 2 h OGTT glucose levels were positively correlated with mean monthly temperatures (r = 0.079 and r = 0.093, respectively)
Wainstock, 2019 [33]	*n* = 59,882, Israel	Retrospective cohort: Included all pregnant women who underwent a GCT from 2005 to 2016 at Central District of Clalit Health Services in Israel.	29.5 ± 51 years	Seasons: Winter (Nov-Mar) Spring (April–May Summer (June-Aug) Autumn (Sept-Oct) Combined: Hot season (summer & spring) Cold season (fall & winter)	1) Glucose levels (GCT & OGTT) 2) Abnormal GCT 3) Abnormal OGTT Thresholds: Carpenter and Coustan 50-g GCT & 100-g OGTT	Maternal age and BMI	•Mean GCT glucose levels and incidence of abnormal GCT varied by season – lowest in winter, followed by spring, fall, and summer •GCTs performed in the winter had the lowest risk of being abnormal (e.g. OR 1.58, 95%CI 1.51. 1.66, for GCT in summer compared to winter) •No significant difference in rate of abnormal OGTT between seasons •Results were similar when comparing warm and cold seasons
Retnakaran, 2018 [41]	*n* = 1464, Toronto, Canada	Cohort: enrolled pregnant women at time of GDM screening	34 ± 4 years	Ambient temperature (°C): 1) mean temp 2) daily temp change. Mean temperature: day of OGTT, 7, 14, 21, 28, 35, 42, 49, 56 days pre-OGTT. Temperature ∆: daily difference between min and max temp on OGTT day and average ∆ 7, 14, 21, 28, 35, 42, 49, 56 days pre-OGTT	1) Glucose levels (OGTT) 2) Insulin resistance (Matsuda index & HOMA-IR) 3) Beta cell function (ISSI-2 & (IGI)/HOMA-IR) 100-g OGTT in late 2nd trimester	Maternal age, ethnicity, FH of diabetes, pp-BMI, GWG through OGTT, week gestation at time of OGTT	•In covariate-adjusted models, temperature ∆ in the pre-OGTT periods (14, 21, 28, 35, 42, 49, 56 days) were positively associated with blood glucose (OGTT fasting glucose & AUC_glucose_) •Temperature ∆ in the pre-OGTT periods (7, 14, 21, 28, 35, 42, 49, 56 days) were inversely associated with ISSI-2 and IGI/HOMA-IR •Mean temperature in the weeks preceding OGTT were suggestively associated with higher OGTT fasting glucose and AUG_glucose_ •Stratified by season: temperature ∆ associated with increased OGTT fasting glucose (e.g. 28 days pre OGTT: β 0.07, *p* = 0.001), AUC_glucose_, and decreased beta cell function, in season where daily temperature is increasing (February – July) Mean temperature associated with increased OGTT fasting glucose (e.g. 28 days pre OGTT: β 0.039, *p* = 0.04), AUC_glucose_ and suggestively decreased beta cell function, only in February–July
Vasileiou, 2018 [27]	A) *n* = 7618 B) *n* = 768, Athens, Greece	Two cohorts: A) Retrospective cohort: 7618 pregnant women who underwent a 100 g OGTT from 2002 to 2012. B) Prospective cohort: 768 pregnant women enrolled in 3rd trimester followed over 18-month period from 1/2013 to 6/2014.	Not provided	A) Season B) Ambient Temperature: 1) Mean monthly temperature 2) Daily temperature (9 am) Three temp groups: 1) < 24.9 °C 2) 25–29.9 °C 3) > 30 °C	Glucose levels (OGTT) Study A: 100 g OGTT in third trimester Study B: 75 g OGTT in third trimester	Maternal age, gestational age, BMI, GWG, blood pressure	Study A: •Blood glucose levels at 1, 2, 3 h differed significantly by season – highest in summer and lowest in winter Study B: •Temperature was positively associated with 1 h glucose levels •Daily temperature > 25 °C was associated with increased risk of abnormal 1 h glucose levels (RR 2.2, 95% CI 1.5, 3.3) •1 h and 2 h glucose levels were significantly higher in > 30 °C daily temperature group
Chiefari, 2017 [42]	*n* = 5473 Calabria, Italy	Cohort: Study population formed based on women who underwent an OGTT for GDM screening at a hospital in Calabria, Italy from 8/2011 to 12/2016.	33 (29–36) years	Seasons: Fall, Winter, Spring Summer, Warm half & cold half of the year, 24-h average temperature each month	Glucose levels (OGTT) 75 g OGTT at 16–18 or 24–28 weeks	Maternal age, ppBMI, prior GDM, FH of diabetes	•Mean 1 h and 2 h glucose levels were highest in summer (1 h, 144; 2 h, 115) compared to other seasons •Fasting glucose levels did not vary by season •Higher 24-h average monthly temperature was associated with increased 1 h and 2 h glucose levels, but not fasting levels
Katsarou, 2016 [25]	*n* = 11,538, Skane county, Sweden	Cohort: Mamma Study, recruited women from 4 obstetric delivery departments in Skane county, Sweden from 2003 to 2005.	29.9 ± 5.1 years	Seasons: Winter (Dec-Feb) Spring (Mar-May) Summer (June-Aug) Fall (Sept-Nov) Mean monthly ambient temperature	Glucose levels (OGTT) 75 g OGTT at 28 weeks (capillary plasma glucose)	Maternal age	•Mean monthly temperature was positively associated with 2 h glucose levels, e.g. 0.009 mmol/L increase in glucose per degree (*p* < 0.001) •OGTT during summer was associated with increased 2 h glucose levels
Moses, 2016 [45]	*n* = 7343, Wollongong, Australia	Cohort: pregnant women with OGTT medical record data from 2012 to 2014, from public and private pathology labs in the Wollongong, Australia area.	Not provided	Seasons: Summer (Dec-Feb) Fall, Winter, Spring	Glucose levels (OGTT) 75 g OGTT at 24–28 weeks, Thresholds: Modified WHO (2006)	Unadjusted	•1 h and 2 h blood glucose were significantly lower in winter compared to the overall mean levels (*p* < 0.0001) •Glucose mmol/L), median (IQR) Winter: 1 h 6.7 (5.0–7.8); 2 h 5.6 (4.8–6.6) Overall: 1 h 6.9 (5.9–8.1); 2 h 5.8 (5.0–6.7)
Janghorbani, 2006 [46]	*n* = 4852, Plymouth, United Kingdom	Cohort: study population based on pregnant women in Plymouth, UK screened for GDM from 1/1996 to12/1997 using data from Plymouth Child Health Database and laboratory and midwifery notes.	GDM: 30.9 ± 5.5 years, Non-GDM: 28.1 ± 5.4 years	Month and season	Glucose levels (OGTT & random plasma glucose) Random plasma glucose followed by 75 g OGTT at 26–28 weeks	Maternal age, birthweight, gestational age	•In covariate-adjusted models, month and season were not associated with glucose levels •OGTT glucose levels did not vary significantly by month or season
Moses, 1995 [26]	*n* = 2749, Wollongong, Australia	Cohort: study population based on women with available OGTT data collected from clinics and obstetric offices from 1/1993 to 6/1994.	27 ± 5.1 years	Month & Season: Summer, Fall, Winter, Spring. Mean monthly ambient temperature (9 am)	Glucose levels (OGTT) 75 g OGTT at mean 28 weeks	Maternal age, parity, BMI, week of testing	•Mean monthly temperature was positively associated with 2 h glucose levels (β 0.026 mmol^− 1^, *p* = 0.01) •2 h glucose levels differed significantly by season – highest in summer and lowest in winter (*p* = 0.011)
Schmidt, 1994 [47]	*n* = 1030, Porto Alegre, Brazil	Cohort: study subjects were women 20+ years receiving prenatal care at two university hospitals with OGTT results during 24–28 weeks gestation from 7/1991 to 3/1993.	20–45 years	Daily ambient temperature (9 am)	1) Glucose levels (OGTT) 2) Abnormal OGTT (≥7.8 mmol/L, 2 h) 75 g OGTT at 24–28 weeks	Maternal age and BMI at enrollment	•Frequency of abnormal glucose tolerance was positively associated with temperature (e.g. 10% at 20–24° vs. 4.9% at 15–19 °C) •1 h and 2 h glucose levels were positively associated with daily temperature (0.07 mmol/L per degree increase in temperature)

*Abbreviations*: *GCT* glucose challenge test, *OGTT* oral glucose challenge test, *GDM* gestational diabetes mellitus, *HOMA-IR* homeostatic model assessment for insulin resistance, *ISSI-2* insulin sensitivity index-2, *IGI* insulinogenic index, *AUC*_*glucose*_ area under the glucose response curve, *WHO* World Health Organization, *BMI* body mass index

#### Climate factors & GDM

##### Seasonality of GDM

Twelve studies evaluated associations between season and GDM (Table 2) [25–27, 37–40, 42, 44–46, 54]. Despite differences in geographical location and seasonal definitions, the majority of studies consistently reported higher incidence or prevalence of GDM diagnosis in the summer and lower incidence or prevalence of GDM diagnosis in the winter [21, 25, 27, 37, 38, 42, 45], while only three studies, two Australian studies (*n* = 2749 [26]; *n* = 2120 [40]) and one in the United Kingdom (*n* = 1074 [39]) reported no association between season of screening and GDM. A population-based study of 4852 pregnancies located in Plymouth, UK, reported higher prevalence of GDM in the spring (2.3, 95% CI: 1.5, 3.2) rather than the summer but consistently lower GDM prevalence in the winter (1.4, 95% CI: 0.8, 2.3) compared to other seasons, although the differences were not statistically significant [46]. In a large South Australian study (*n* = 60,306), incidence of GDM was significantly associated with season of estimated date of conception, where adjusted incidence of GDM was highest in pregnancies with estimated conception dates in August (6.6%) (Australian winter), with women entering second trimester—the time period of increasing insulin resistance—during the months of increasing temperature [44]. The lowest prevalence of GDM was among pregnancies with estimated conception dates in January (5.4%) (Australian summer) [44], with women entering second trimester during the months of decreasing temperatures.

##### Seasonality of glycemic outcomes

As with GDM, seasonal variations in gestational glucose levels have been reported as early as 1995 [26]. Eleven studies assessed the relationship between seasonality and maternal glycemic outcomes (Table 3) [25–27, 33, 37–40, 42, 45, 46]. Associations of season with blood glucose levels have been somewhat less consistent across studies compared to those with GDM, but studies have generally reported higher glucose levels in the summer. Two UK-based studies, one population-based study in Plymouth, UK (*n* = 4852) and one smaller cohort in Cambridge, UK (*n* = 1074) reported no associations of month or season of screening with glucose levels from fasting 2-h 75-g OGTTs [39, 46]. A much larger Israeli retrospective cohort study (*n* = 59,882) also reported no associations of season of screening with glucose levels from a fasting 3-h 100-g OGTT [33]. However, the authors did observe lower glucose levels from a non-fasting 50-g GCT in winter and higher GCT glucose levels in summer, as well as significantly higher odds of elevated GCT glucose levels (> 140 mg/dL) in summer compared to winter (OR 1.58, 95% CI: 1.51, 1.66) [33]. Five studies in Italy (5473) [42], Greece (*n* = 7618) [27], Spain (*n* = 2366) [38], and two in Australia (*n* = 7343, [45]; *n* = 2120, [40]), observed higher 1 h and 2 h glucose levels from 2-h 75-g (3-h 100-g, [27, 38]) OGTTs in the summer and/or lower levels in the winter (autumn, [38]) compared to other seasons. Two studies in Sweden (*n* = 11,538) [25] and Australia (*n* = 2749) [26] reported higher 2 h glucose levels from a 75-g OGTT in the summer, but did not see differences in 1 h glucose levels by season [25, 26]. However, the majority of these studies did not find variations in fasting glucose by season, except for a cohort in Australia that reported lower fasting glucose levels in summer compared to winter [40], which may reflect different regulation or sensitivity compared to postprandial glucose levels.

##### Ambient temperature & GDM

Eight studies evaluated the association between ambient temperature at varying time points during pregnancy and GDM (Table 2) [21, 25–27, 37, 38, 41, 43]. Three studies found no association between various measures of ambient temperature and GDM [25–27]. An older Australian study (n = 2749) [26] and a larger Swedish cohort study (*n* = 11,538) [25], reported no association between mean monthly temperature during the month of glucose screening and GDM, and a small Greek study (*n* = 768) reported no association between daily temperature at time of glucose screening and GDM [27]. The remaining five studies reported associations of various ambient temperature variables with increased odds or risk of GDM [21, 37, 38, 41, 43].

Three studies reported associations of mean temperature on the day of OGTT screening with increased odds or risk of GDM [21, 37, 38]. Mean temperature in the days and weeks leading up to the OGTT was also associated with increased odds or risk of GDM in some studies [21, 38, 43]. For example, a large Canadian registry-based cohort of 555,911 pregnancies from the greater Toronto area, higher ambient temperature averaged over the 30-days preceding routine GDM screening (~ 27 weeks gestation) was significantly associated with higher odds of GDM [43]. Specifically, each 10-degree increase in mean 30-day temperature was associated with a 6% increased odds of GDM [43]. A separate smaller Toronto-based cohort study (*n* = 1464), found no association between mean ambient temperature and GDM when outdoor temperature was modeled as mean temperature in the weeks prior to GDM screening (i.e. 7, 14, 21, 28, 35, 42, 49, 56 days pre-screening) [41]. However, researchers did see associations between average daily change in ambient temperature (defined as the difference between the daily minimum and maximum temperatures) over the weeks pre-GDM screening and increased odds of GDM. For example, the odds of GDM were 1.20 (95% CI 1.05, 1.37) per degree increase in average daily change in ambient temperature (°C) over the 14 days pre-GDM screening, with similar results over different time windows. These associations were only seen in the seasons where daily temperatures were increasing (February–July, i.e. Spring-Summer) not in the seasons where daily temperatures were decreasing (August–January, i.e. Fall-Winter) [41]. Similarly, a Spanish cohort reported associations between higher mean temperatures on the day of the OGTT as well as 14- and 28-days pre-OGTT and increased risk of GDM (e.g. mean temp day of OGTT: OR 1.029, 95% CI: 1.005–1.054), but in stratified analyses these associations were present only during seasons where daily temperatures were increasing [38], which may indicate the importance of other seasonal and physiological factors such as acclimatization, beyond ambient temperature alone.

Of note, all three of the studies reporting no association between temperature and GDM used 1-step screening approaches to diagnose GDM (Tables 1 & 2), whereas the five studies that reported positive associations between temperature variables and GDM reported using a 2-step screening approach or were located in countries where a 2-step screening approach is most commonly used [55], although all women underwent both a GCT and 3 h OGTT as part of the study protocol in Retnakaran et al. [41] and both the IADPSG and Carpenter and Coustan criteria are used in Taiwan [21].

##### Ambient temperature & glycemic outcomes

Eight studies assessed associations of ambient temperature measures with maternal glycemic outcomes, including glucose levels as continuous outcomes, abnormal glucose screening results, insulin resistance, and beta cell function (Table 3) [25–27, 38, 40–42, 47]. Three cohorts, one in Greece (*n* = 768) [27], one in Brazil (*n* = 1030) [47], and one in Spain (*n* = 2366) [38] found associations between higher ambient temperatures measured on the day of glucose screening (fasting 2-h 75-g OGTT [27, 47]; 3-h 100-g OGTT [38]) and higher 1 h and 2 h OGTT glucose levels. In four larger cohorts from Sweden (*n* = 11,538) [25], Italy (*n* = 5473) [42], and two from Australia (*n* = 2749 [26], mean temperature of the calendar month of glucose testing (fasting 2-h 75-g OGTT) was positively associated with 2 h OGTT glucose levels, but only with 1 h OGTT glucose levels in two of the cohorts [40, 42].

In contrast, a recent Canadian study (*n* = 1464) only found suggestive covariate-adjusted associations of measures of mean temperature in the weeks prior to glucose testing (i.e. 7, 14, 21, 28, 35, 42, 49, 56 days pre-screening) with higher glucose levels from a fasting 3-h 100-g OGTT [41]. However, they observed stronger and significant positive associations of mean daily temperature change in the weeks prior to glucose testing (defined as the difference between the daily minimum and maximum temperatures) with both OGTT fasting glucose levels and AUC_glucose_ (fasting, 30, 60, 120, and 180 min) in covariate-adjusted models [41]. Increased mean daily temperature change in the weeks prior to glucose testing was also associated with decreased beta cell function based on measures of ISSI-2 and (IGI)/HOMA-IR. When stratified by season, they found that these associations were only present in the season where daily temperatures were increasing (February – July, i.e. Spring-Summer) and not in the season where daily temperatures were decreasing (August – January, i.e. Fall-Winter) [41], indicating the potential importance of other seasonal factors, such as magnitude and direction of temperature fluctuations, rather than simple static temperature. Similar to results of the seasonality studies, the majority of the above studies found no association between temperature and fasting glucose levels, however two recent studies found inverse associations between temperature and fasting glucose levels [38, 40].

## Discussion

In this systematic review, we summarized the current epidemiologic evidence evaluating climate factors and GDM. We found consistent evidence for an association of the summer season with GDM incidence or prevalence [21, 25, 27, 37, 38, 42, 44–46] and 2 h glucose levels on the OGTT [25–27, 38, 40, 42, 45]. Evidence of an association between temperature and GDM was less consistent across studies. However, despite some heterogeneity in study findings, collective results suggest that higher ambient temperatures may be associated with higher glucose levels from GDM screening tests [25–27, 38, 40, 41, 47] and higher odds or risk of GDM [21, 37, 38, 41, 43]. Current study findings suggest that climate factors may be important to consider in the study and prevention of GDM, especially in the context of current and projected global climate change.

The consistent association of season with glycemic outcomes is supported by findings that season is associated with diabetes risk outside of pregnancy. Seasonal patterns have been observed for type 1 diabetes incidence [30]. However, unlike GDM incidence, which peaks in the summer, incidence of type 1 diabetes diagnosis generally peaks in the winter months. Type 1 diabetes and GDM have different pathophysiology, which may explain the discrepancy in seasonal patterns between diseases. Glycemic control among individuals with type 2 diabetes also varies by season in a similar pattern as with type 1 diabetes, with winter months showing higher hemoglobin A1c levels [56, 57]. Conversely, increased ambient temperature and heat stress have been consistently associated with increased risk of diabetes (type 1 and 2) and glucose intolerance in non-pregnant populations, supporting the association between temperature and increased glucose levels and GDM odds/risk during pregnancy [29]. For example, a large Spanish cohort of non-pregnant adults reported positive associations between higher mean annual ambient temperature and higher prevalence of prediabetes, diabetes, and insulin resistance, as well as higher fasting and 2-h glucose levels and HOMA-IR [58]. The fact that season is associated with the incidence of type 1 diabetes, glycemic control in type 2 diabetes, and GDM, coupled with the relatively consistent findings of effects of ambient temperature on diabetes odds/risk and glucose intolerance in pregnant and non-pregnant populations, suggest the need for future studies of seasonality and temperature related to diabetes risk, with a particular focus on the mechanisms and reasons for the discordant seasonal results across diabetes types.

The studies included in this review varied considerably in terms of exposure assessment, of GDM diagnostic criteria, glucose measurement, methodological approaches, and geographic location, which may explain some of the heterogeneity in study results. Results from the included studies on ambient temperature and GDM were inconsistent, which may partially be due to differences in GDM screening methods across studies (Table 1). Different diagnostic criteria across studies can affect estimated GDM prevalence, and could affect observed associations between climate factors and GDM. While the studies that found no association between ambient temperature and GDM all utilized 1-step diagnostic criteria with 2 h 75-g OGTTs, the studies that reported associations between temperature variables and GDM reported using a 2-step diagnostic approach and/or were located in countries where a 2-step approach is generally used, although one study administered OGTTs to all women, and 3 h 100-g OGTTs were administered. Differences in glucose screening method (e.g. 75-g vs. 100-g OGTT) or timing of testing during pregnancy, could also contribute to the inconsistencies observed between associations of ambient temperature or season with different OGTT glucose levels. Some studies reported associations for certain OGTT glucose levels but not others (e.g. 2 h but not the 1 h values) [25, 26], while another study reported associations with the GCT glucose levels, but not with OGTT glucose levels [33]. Differences in ambient temperature patterns and ranges across geographic regions could also have contributed to inconsistencies in study findings. GDM prevalence is highly variable across geographic regions and is highest in the Middle East (median 15.2%) and South-East Asia (15.0%), regions with extreme high temperatures, and noticeably lower in Europe (6.1%) and North America (7.0%), regions with relatively lower temperatures [59]. However, no studies have investigated associations of absolute ambient temperatures and GDM across geographic regions.

Pregnant women may be more susceptible to the adverse health effects of climate related factors including heat and cold stress [60] and may therefore be particularly vulnerable to the development of diabetes and altered glycemic outcomes associated with climate factors in the face of increases in extreme temperatures.

Multiple hypotheses exist for the observed effect of ambient temperature and season on GDM and glycemic outcomes. One possible mechanism is through brown adipose tissue activation, which can induce weight changes and insulin sensitivity [29, 61, 62]. A recent study reported a positive association between ambient temperature and glycated hemoglobin (HbA1c) levels in 65,535 clinical patients [63]. The researchers hypothesized that these fluctuations could be due to seasonal alterations in brown adipose tissue (BAT) and its activation. BAT is sensitive to temperature fluctuations. At temperatures below the thermoneutral zone (i.e. 27–30 °C in unclothed adults), BAT is activated to produce heat [64], consuming large amounts of glucose from the blood in the process [65–68]. Glucose uptake during non-shivering thermogenesis in BAT occurs through upregulation of mitochondrial uncoupling protein 1 (UCP1) and glucose transporters (GLUT1 and GLUT4) [63, 69, 70]. Additionally, heat shock protein (HSP) expression in BAT is induced at cold temperatures [71–73]. Patients with diabetes and insulin resistance have reduced Hsp70 expression [74]. Reduced Hsp70 has been associated with hyperglycemia during pregnancy in mice [75].

In rodents, BAT may be especially important in regulating glucose metabolism during pregnancy; Qiao et al. showed that fetuses of UCP1 knockout dams had significantly higher blood glucose levels than wild-type dams [76]. At higher temperatures, there is a decrease in temperature-induced BAT activation, which could result in a decrease in glucose uptake. Separately, at higher temperatures beige and brown adipocytes can shift in chromatin structure to resemble white adipose tissue, leading to reduced expression of UPC1 and down regulation of lipid metabolism pathways [75]. In a clinical human population, BAT, detected using positron-emission tomographic and computed tomographic (PET-CT) scans, was inversely correlated with ambient temperature on the day of scan [77].

Alternative hypotheses to explain the positive association between ambient temperature and glucose levels include increased beta-cell dysfunction, insulin resistance, hemoconcentration and increased arterialization of venous blood [5, 33, 78]. One study in our review looked at associations between ambient temperature and markers of beta-cell function. The authors found that the higher mean daily temperature change in the 3–5 weeks prior to OGTT screening was significantly associated with decreased ISSI-2 and (IGI)/HOMA-IR, implicating beta-cell dysfunction as a potential pathophysiological mechanism behind the parallel associations with higher glucose levels and GDM prevalence, although suggestive associations were also seen with increase insulin resistance (i.e. Matsuda Index and HOMA-IR) [41]. In a large Spanish cohort of non-pregnant adults, higher annual mean temperature was associated with increased insulin resistance (HOMA-IR) [58]. Alternatively, higher ambient temperatures and increased activity in warmer weather could lead to dehydration, and resulting hemoconcentration, which could cause an apparent increase in blood glucose concentrations during glucose screening and resulting GDM diagnoses [33]. Similarly, blood flow distribution is altered at high temperatures, leading to increased arterialization of venous blood, which could result in higher glucose levels measured in venous blood samples leading to increases in failed GDM screening tests and GDM diagnoses. In climate chamber experiments, Moses et al. [5] and Dumke et al. [78] observed significantly increased levels of OGTT glucose levels [5, 78] and insulin levels [78] at higher temperatures in healthy male subjects. These physiological changes at high temperatures could cause transient elevations in measured blood glucose levels, leading to potential misclassification of abnormal glucose tolerance and GDM.

Seasonal factors unrelated to temperature may also contribute to the observed seasonal variation in GDM. Circadian and thermogenic networks in BAT are controlled by the nuclear receptor Rev-erbα, which represses UPC1 expression in BAT [79, 80]. Consequently, studies have shown that UCP1 expression in BAT follows a circadian rhythm in rodents, peaking during the day [63]. Seasonal changes in circadian rhythms, could contribute to the observed seasonal patterns of GDM risk. Alternatively, seasonal variations in physical activity and dietary patterns, as well as access to green space may also play a role in the observed seasonal variation in GDM and glycemic outcomes. Vitamin D may help regulate glucose homeostasis by increasing insulin sensitivity and beta-cell function, or decreasing systemic inflammation [35] and lower Vitamin D levels and Vitamin D deficiency, have been associated with increased GDM risk [81–85] and higher fasting glucose and insulin resistance during pregnancy [85]. Decreased sun exposure in winter months during early pregnancy could lead to decreased vitamin D levels during early pregnancy, which has been associated with subsequent risk of GDM during later pregnancy, at the time of routine GDM screening [82] In addition to increased GDM risk, lower Vitamin D levels during early pregnancy have been associated with higher HOMA-IR, lower Matsuda index, and lower ISSI-2 during in later pregnancy (24–28 weeks) [82].

Temperature and season also affect other environmental factors known to be associated with GDM, such as air pollution [23, 24]. For example, exposure to ambient air pollutants, including nitrous oxides and PM_2.5_ (i.e. particulate matter with a diameter of 2.5 μm or less), has been consistently associated with increased GDM risk [84, 86–91]. Additionally, potential seasonal changes in gut microbiome could contribute to the observed seasonal variation in glycemic outcomes [92, 93]; in GDM complicated pregnancies, altered maternal gut microbiome have been reported [94, 95]. Overall, the exact mechanism behind the effect of season and ambient temperature on GDM and glycemic outcomes remains unclear. Further research is needed to investigate the interplay between temperature and additional meteorological factors with glucose regulation in pregnancy using toxicologic and epidemiologic studies.

None of the identified studies assessed the associations of other meteorological-related factors such as precipitation and humidity on GDM risk. Seasonal variations in these factors could also contribute to the observed variation in GDM and glycemic outcomes. For example, a recent study found a positive correlation between diabetes incidence and precipitation in Cameroon [96]. Exposure to ambient air pollution has also been associated with increased risk of GDM [23] and varies by season and meteorological factors. However, further research is needed to understand the role of these additional factors and how they may be related to or interact with temperature to impact risk of GDM.

There is growing evidence that certain subsets of the population (i.e. those with a family history of diabetes or individuals who are overweight/obese) may be more susceptible to the effects of environmental exposures compared to others [97–100]. However, none of the reviewed studies assessed effect modification of the associations between climate factors and GDM or maternal glycemic outcomes by factors such as family history of diabetes, pre-pregnancy BMI, age, or race/ethnicity. Future studies should assess heterogeneity in associations across different population subgroups.

Due to the ubiquitous nature of environmental exposures, individuals are continuously exposed to complex combinations of environmental factors. While there is increasing awareness for the need to incorporate analyses of exposure mixtures and combinations in epidemiologic studies, none of the current studies have looked at cumulative effects of environmental exposures in the context of multiple climate-related factors such as temperature, humidity, and precipitation. Further research is needed to understand the potential cumulative and interactive effects of these climate factors on GDM risk.

Strategies for Evaluating Climate Factors in Future Studies of GDM:

1. Consider evaluating climate factors and accounting for lag time in modeling procedures.
2. Standardize GDM diagnostic criteria and screening methods.
3. Evaluate possible heterogeneous effects of climate factors across population subgroups by assessing effect modification to identify particularly vulnerable populations.
4. Consider the impact of climate factors on various populations based on region and space and the differential effects in given geographic areas.
5. Evaluate the potential confounding and/or modifying role of season of GDM diagnosis and ambient temperature prior to GDM screening in studies of pregnancy and neonatal outcomes in GDM complicated pregnancies.
6. Evaluate the cumulative impact of multiple climate-related factors (e.g. temperature, season, humidity, precipitation, and the interplay with air pollution and other environmental factors) on GDM and related outcomes.

## Conclusion

In summary, there is mounting evidence that exposure to certain climate factors—ambient temperature and season—during pregnancy is associated with increased risk of developing GDM and adverse glycemic outcomes. The seasonality of GDM was consistent across studies, with higher prevalence of GDM generally observed in the summer months. Furthermore, higher ambient temperature may be associated with elevated glucose levels from GDM screening tests. Associations between ambient temperature and GDM were more inconsistent; however, this could be due to differences in GDM diagnostic criteria. One major limitation of the current literature is the lack of consistency in both exposure and outcome assessment across studies; future studies should work to standardize these methods. Furthermore, future work should include more diverse study populations to allow researchers to identify potential high-risk population subgroups. Finally, future studies should include an emphasis on evaluating effects of exposure to multiple environmental and climate factors. Given the current GDM epidemic coupled with current and projected global climate change, understanding the extent to which climate factors might affect GDM risk is imperative to reducing the risk of this increasingly prevalent and costly pregnancy complication.

## Abbreviations

GDM: Gestational diabetes mellitus
BMI: Body mass index
PRISMA: Preferred reporting items for systematic reviews and meta-analyses
GCT: Glucose challenge test
OGTT: Oral glucose tolerance test
IADPSG: International Association of Diabetes in Pregnancy Study Group
IR: Insulin resistance
HOMA-IR: Homeostasis model assessment of insulin
ISSI-2: Insulin sensitivity index 2
IGI: Insulinogenic index
WHO: World Health Organization
ADIPS: Australian Diabetes in Pregnancy Society
NDDG: National Diabetes Data Group
FH: Family history
ppBMI: Pre-pregnancy body mass index
GWG: Gestational weight gain
LMP: Last menstrual period
AUC_glucose_: Area under the glucose response curve
PM2.5: Particulate matter with a diameter of 2.5 μm or less
HbA1c: Glycated hemoglobin
BAT: Brown adipose tissue
UCP1: Mitochondrial uncoupling protein 1
GLUT: Glucose transporters
HSP: Heat shock protein
PET-CT: Positron-emission tomographic and computed tomographic
